# In-Depth Molecular Characterization of *Mycobacterium tuberculosis* from New Delhi – Predominance of Drug Resistant Isolates of the ‘Modern’ (TbD1^−^) Type

**DOI:** 10.1371/journal.pone.0004540

**Published:** 2009-02-23

**Authors:** Ruth Stavrum, Vithal Prasad Myneedu, Virendra K. Arora, Niyaz Ahmed, Harleen M. S. Grewal

**Affiliations:** 1 The Gade Institute, Section for Microbiology and Immunology, University of Bergen, Bergen, Norway; 2 Department of Microbiology and Immunology, Haukeland University Hospital, Bergen, Norway; 3 Lala Ram Sarup Institute of Tuberculosis and Respiratory Diseases, New Delhi, India; 4 Pathogen Biology Laboratory, Department of Biotechnology, University of Hyderabad, Hyderabad, India; University College Dublin, Ireland

## Abstract

**Background:**

India has the highest estimated burden of tuberculosis in the world, accounting for 21% of all tuberculosis cases world-wide. However, due to lack of systematic analysis using multiple markers the available information on the genomic diversity of *Mycobacterium tuberculosis* in India is limited.

**Methodology/Principal Findings:**

Thus, 65 *M. tuberculosis* isolates from New Delhi, India were analyzed by spoligotyping, MIRU-VNTR, large deletion PCR typing and single nucleotide polymorphism analysis (SNP). The Central Asian (CAS) 1 _DELHI sub-lineage was the most prevalent sub-lineage comprising 46.2% (n = 30) of all isolates, with shared-type (ST) 26 being the most dominant genotype comprising 24.6% (n = 16) of all isolates. Other sub-lineages observed were: East-African Indian (EAI)-5 (9.2%, n = 6), EAI6_BGD1 (6.2%, n = 4), EAI3_IND, CAS and T1 with 6.2% each (n = 4 each), Beijing (4.6%, n = 3), CAS2 (3.1%, n = 2), and X1 and X2 with 1 isolate each. Genotyping results from five isolates (7.7%) did not match any existing spoligopatterns, and one isolate, ST124, belonged to an undefined lineage. Twenty-six percent of the isolates belonged to the TbD1+ PGG1 genogroup. SNP analysis of the *pncA* gene revealed a CAS-lineage specific silent mutation, S65S, which was observed for all CAS-lineage isolates (except two ST26 isolates) and in 1 orphan. Mutations in the *pncA* gene, conferring resistance to pyrazinamide, were observed in 15.4% of all isolates. Collectively, mutations in the *rpoB* gene, the *katG* gene and in both *rpoB* and *katG* genes, conferring resistance to rifampicin and isoniazid, respectively, were more frequent in CAS1_DELHI isolates compared to non-CAS_DELHI isolates (OR: 3.1, CI95% [1.11, 8.70], P = 0.045). The increased frequency of drug-resistance could not be linked to the patients' history of previous anti-tuberculosis treatment (OR: 1.156, CI95% [0.40, 3.36], *P* = 0.79). Fifty-six percent of all new tuberculosis patients had mutations in either the *katG* gene or the *rpoB* gene, or in both *katG* and *rpoB* genes.

**Conclusion:**

CAS1_DELHI isolates circulating in New Delhi, India have a high frequency of mutations in the *rpoB* and *katG* genes. A silent mutation (S65S) in the *pncA* gene can be used as a putative genetic marker for CAS-lineage isolates.

## Introduction

The Indian subcontinent has been a global hotspot for the growth and spread of the TB epidemic in recent times [1] and has served as the corridor of early world-wide dissemination of *M. tuberculosis* during the ancient era [2]. *Mycobacterium tuberculosis* genotypes from the Indian subcontinent have largely been described using, in most cases, single techniques to define bacterial diversity [3]–[7]. Such studies, although important, underestimate the clonal diversity of *M. tuberculosis*. IS*6110* restriction-fragment length polymorphism (RFLP) has been the gold standard for population-based molecular epidemiological studies of TB with the purpose of identifying pathways and predictors of ongoing TB transmission [8], [9]. However, some *M. tuberculosis* isolates, especially those belonging to ancient lineages, have few or no IS*6110* copies [10], [11]. Moreover, systematic fingerprinting of all *M. tuberculosis* isolates by IS*6110* RFLP is time-consuming, cumbersome and the results have poor inter-laboratory portability [10], [12]. Thus, a combination of two rapid PCR-based molecular typing methods; spoligotyping and variable number tandem repeats of mycobacterial interspersed repetitive unit (MIRU-VNTR) has become an attractive alternative to IS*6110* RFLP. A study by Hanekom *et al* [13] suggests that there are discriminatory limitations to the available genotyping methods in high TB-incidence areas and that the choice of appropriate MIRU-VNTR loci require further investigation in diverse *M. tuberculosis* lineages in low- and high TB-endemic countries. A few recent studies that have analyzed tubercle bacilli from India using multiple markers [2], [14], [15] reveal a clear predominance of two important genogroups (TbD1^−^ and TbD1^+^ groups of strains), confined to North and South India, respectively. While the TbD1^+^ East African Indian (EAI) genofamily and the TbD1^−^ hypervirulent and multidrug-resistant (MDR) -associated Beijing strains predominates in the southern part of the country, the TbD1^−^ Delhi types, which, by spoligotyping are characterized by lack of at least spacers 4–7 and 23–34 [16] are overwhelmingly represented in Delhi and its adjoining states [7], [17], [18]. While EAI genotypes have been dissected at genomic levels [14], no studies till date have been performed to investigate the genetic composition of the Delhi type/Central Asian (CAS) 1 isolates. Our study therefore, aimed at analyzing the genetic makeup of circulating *M. tuberculosis* isolates in metropolitan Delhi with an in-depth characterization of major lineages and sub-lineages.

## Materials and Methods

### Patients and isolates

Following study approval by the Ministry of Health (reference: V.21011/20/2002-CCD), India and the Lala Ram Sarup (LRS) Institute of Tuberculosis and Respiratory Diseases, New Delhi, isolates were obtained from pulmonary tuberculosis (TB) patients attending the TB clinic of the LRS Institute. Sputum samples were collected as part of the routine diagnostic examination of suspected pulmonary TB patients at LRS. Verbal consent for analysis of *M. tuberculosis* isolates from sputum samples were obtained from all patients. Additionally, as part of the routine clinical examination at the LRS Institute, information on current and previous history of *M. tuberculosis* infection, history of contact with a TB case and a history of receiving previous anti-tuberculosis treatment was available. None of the study participants could be identified based on the analyses undertaken on the *M. tuberculosis* isolates analyzed in this study. Of 100 culture-positive acid-fast isolates, 23 isolates were not viable upon sub-culturing. Of 77 isolates that were available for this study, 9 were by the Hain's kit (Hain Lifescience, Nehren, Germany) and 16S rRNA sequencing found to be atypical mycobacteria and excluded from further analyses. Thus, isolates from 68 patients were included in this study. Forty-eight percent of the patients included were male and the mean age was 30.6 years with 84.6% of the patients <45 years of age. Twenty-four (38.1%) patients were previously treated for TB.

### DNA isolation

Chromosomal DNA was isolated as described by van Embden *et al* [9] or by heat-extraction using a loopful of cells suspended in 200 µl of TE buffer (10 mM Tris-Cl, 1 mM EDTA), and heat-killed by incubation at 95°C for 20 min. The supernatant containing the extracted DNA was collected by centrifugation at 12000 rpm for 10 min.

### Spoligotyping

Spoligotyping was performed with commercially available activated Biodyne C membranes with the 43 synthetic oligonucleotides covalently bound to the membranes (Isogen Bioscience BV, Maarssen, The Netherlands), as recommended by the manufacturer. The hybridization patterns were converted into binary and octal formats according to Dale *et al* [19] and compared with previously reported strains in the SpolDB4 [16] database.

### MIRU-VNTR analysis

Isolates were genotyped by MIRU-VNTR by amplification of 15 loci as described by Supply *et al* [20]. PCR amplification was performed in a total volume of 20 µl containing 1 µl DNA, 0.04–0.4 µM of all 15 primer sets, and Hotstart *Taq* Plus polymerase Master Mix (Qiagen). All reactions were subjected to 95°C for 5 min, followed by 30 cycles of 30 sec at 95°C, 1 min at 55°C, 1.5 min at 72°C and terminated by 7 min at 72°C. Genotyping was performed using multiplex PCR with a Rox-labeled MapMarker 1000 size standard (PE Applied Biosystems) for sizing of the PCR products. The PCR fragments were analyzed using a capillary-based electrophoresis sequencer (ABI 3700), and sizing of the various VNTR alleles were done using the Peak Scanner Software v1.0 (PE Applied Biosystems). The number of repeats present in each locus was determined and alleles were assigned numerical values accordingly. Furthermore, isolates with identical MIRU-VNTR genotype were defined as belonging to the same cluster.

### Single Nucleotide Polymorphisms

Isolates were assigned to Principal Genetic Groups (PGG) after PCR and subsequent SNP analysis of *katG^463^* and *gyrA^95^* using primers as described in Sreevatsan *et al* [21] (Table 1). Other mutations in the *rpoB*, *embB*, *katG*, *pncA* and *gyrA* genes of the *M. tuberculosis* isolates were determined by PCR and sequencing using primers listed in Table 1. PCR was performed in 20 µl reactions containing 12.5 µl Taq Polymerase (GoTaq Mastermix, Promega), 0.04 µM of each primer, 9.5 µl H_2_O and 1 µl DNA. The PCR reaction consisted of an initial 2 min for denaturation at 95°C, 30 cycles of 95°C for 1 min, 55–65°C, for 1 min, 72°C for 2 min and elongation time of 72°C for 5 min. Sequencing reaction consisted of an initial 5 min for denaturation at 96°C, 25 cycles of 96°C for 15 sec, 50°C for 10 sec, 60°C for 4 min and elongation time of 72°C for 15 min using the BigDye 3.1 Terminator Cycle Sequencing Kit (Applied Biosystems Inc). The sequencing was carried out in an ABI 377 automatic DNA sequencer (Applied Biosystems Inc., Foster, CA) and nucleotide sequences were analyzed using DNASTAR software (DNASTAR, Inc., USA).

**Table 1:** Primers used for the analysis of single nucleotide polymorphisms in 65 *M. tuberculosis* isolates from New Delhi, India.

Gene	Forward (5′->3′)	Reverse (5′->3′)	References
*pncA*	GTCGGTCATGTTCGCGATCG	GCTTTGCGGCGAGCGCTCCA	[43]
*katG*	CATGAACGACGTCGAAACAG	CGAGGAAACTGTTGTCCCAT	[45]
*rpoB*	GGTCGGCATGTCGCGGATGG	GCACGTCGCGGACCTCCAGC	[46]
*embB*	CGGCATGCGCCGGCTGATTC	TCCACAGACTGGCGTCGCTG	[47]
*katG* ^463^	CGAGGAATTGGCCGACGAGTT	CGGCGCCGCGGAGTTGAATGA	[21]
*gyrA* ^95^	CAGCTACATCGACTTGCGA	GGGCTTCGGTGTTACCTCAT	[21]
TbD1 flanking	CTACCTCATCTTCCGGTCCA	CATAGATCCCGGACATGGTG	[22]
TbD1 internal	CGTTCAACCCCAAACAGGTA	AATCGAACTCGTGGAACACC	[22]

An isolate was defined as being MDR if mutations in both the *rpoB* and *katG* genes were observed.

### TbD1 analysis

The presence or absence of TbD1 was analyzed by PCR using 2 primer sets complementary to the sequences of the deleted region or complementary to the internal sequences of the intact region [22]. Isolates containing the TbD1 region (TbD1+) produced a PCR product using the internal primers, whereas isolates that lacked the TbD1 region (TbD1−) produced a PCR product using the flanking primers only.

### Cluster analysis

All results from this study were entered into the Bionumerics program (Bionumerics version 3.1, Applied Maths, Saint-Martens-Latem, Belgium). A dendrogram of the MIRU-VNTR profiles were produced following Pearson correlation and unweighted pair group method with arithmetic average analysis.

### Statistical analysis

The Hunter-Gaston discriminatory index (HGDI) was calculated for each of the 15 MIRU-VNTR loci as described previously [23]. The discriminatory nature of each loci were, based on HGDI values, considered as highly >0.6, moderately 0.3–0.6 and poorly <0.3 as suggested by Sola *et al.* [24]. Differences between proportions were analyzed using the chi-square exact test (with Yates' correction for continuity) and expressed as odds ratios (ORs) with 95% confidence interval (95%CI). The patient's history of previous anti-TB treatment was taken into consideration when assessing a link between mutations conferring anti-TB drug-resistance and the CAS1_DELHI sub-lineage isolates using simple and multivariable logistic regression analyses using SPSS version 14.0 (Softonic). A *P* value of <0.05 was taken to represent statistical significance.

## Results

### MIRU-VNTR

**Figure 1:**
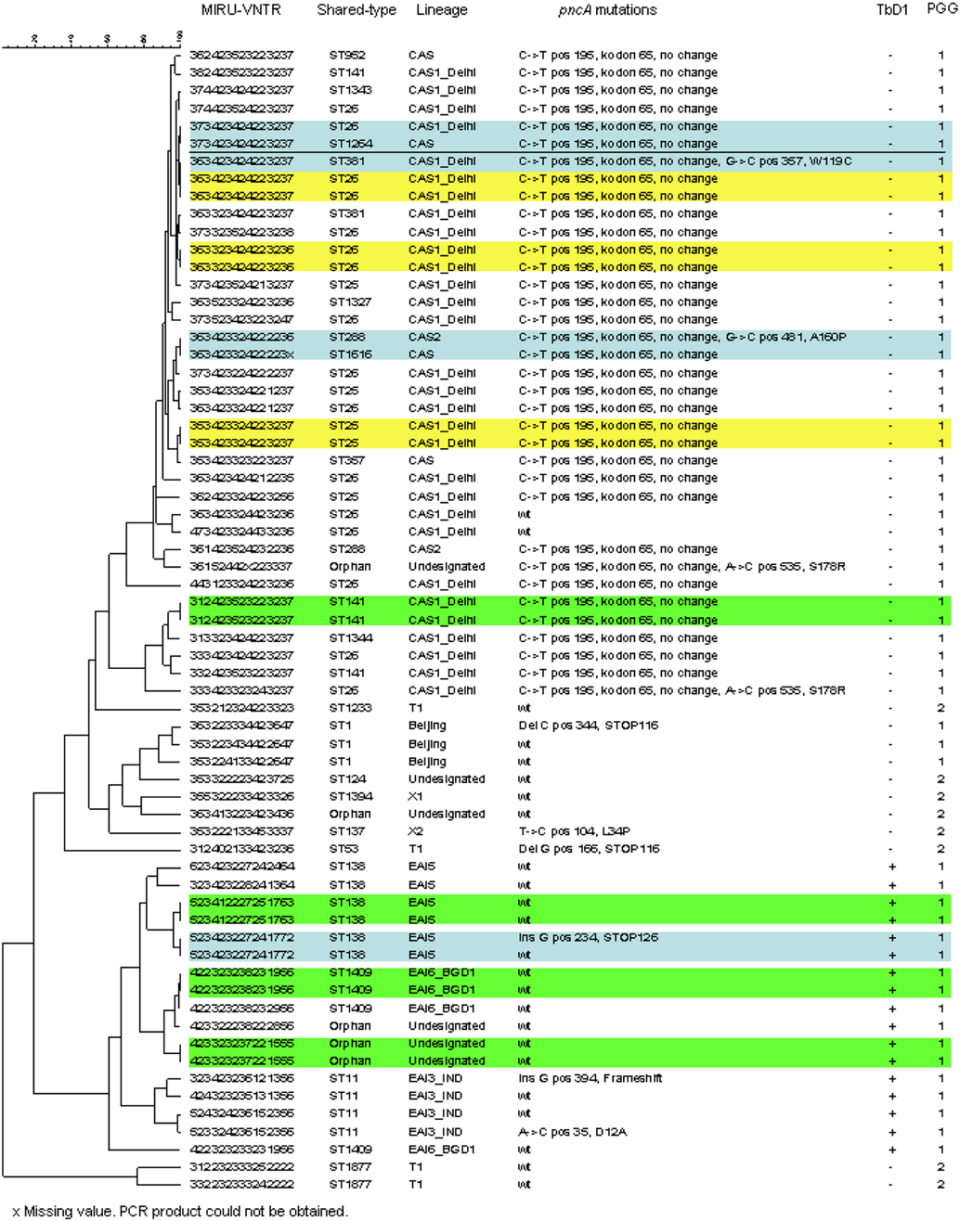
Diversity of 65 *M. tuberculosis* isolates from New Delhi, India deduced from MIRU-VNTR, spoligotyping and single nucleotide polymorphism analyses. MIRU-VNTR clusters that could be discriminated by spoligotyping are highlighted in blue and clusters that could be discriminated based on mutations in the *katG*, *rpoB* and/or *embB* genes are highlighted in yellow. Clusters that could not be discriminated using a combination of all markers are highlighted in green. The dendrogram was produced following Pearson correlation and unweighted pair group method with arithmetic average analysis.

Of the 68 isolates analyzed by MIRU-VNTR, 3 isolates were suspected to be of mixed *M. tuberculosis* sub-populations and were excluded from further analysis. Of the remaining 65 isolates, 21 isolates could, by MIRU-VNTR, be distributed into 10 clusters. Three clusters comprising 6 isolates could be discriminated by SNP analysis of *katG*/*rpoB* genes. Two clusters comprising 2 isolates each could be further discriminated by spoligotyping, whereas in one cluster comprising 3 isolates, two isolates of the same genotype were discriminated by SNP analysis of *rpoB*/*embB* genes. The third isolate in this cluster belonged to a different shared-type (Figure 1). Eight isolates comprising 4 clusters could not be discriminated by a combination of MIRU-VNTR and SNP analyses of *katG*, *rpoB*, *embB*, or *pncA*. No epidemiologic link could be established between the patients. MIRU26 was the most discriminatory locus for isolates belonging to the CAS1_DELHI sub-lineage with a HGDI value of 0.788506, whereas QUB-26 was the most discriminatory locus for EAI5 isolates with a HGDI of 0.8. MIRU-VNTR discriminated only moderately between isolates of the EAI6_BGD1 sub-lineage with ETR-A and QUB-4156c giving the highest HGDI value of 0.5.

### Spoligotyping

Sixty-five isolates from New Delhi were analyzed by spoligotyping and the results compared to the shared-types (ST) and lineages/sub-lineages described in SpolDB4 [16]. Of the 65 isolates with single *M. tuberculosis* sub-population, 60 were assigned to 21 previously described ST's belonging to 10 different sub-lineages (Table 2). CAS1_DELHI (n = 30) was the most dominant sub-lineage and ST26 (CAS1_DELHI) the most frequent genotype (24.6%, n = 16). Other sub-lineages observed in this study were EAI5 (9.2%, n = 6), EAI6_BGD1 (6.2%, n = 4), EAI3_IND (6.2%, n = 4), CAS (6.2%, n = 4), T1 (6.2%, n = 4), Beijing (4.6%, n = 3), CAS2 (3.1%, n = 2), and 2 isolates belonged to X1 and X2, respectively. Five isolates (7.7%) were not described previously and were termed orphans, and 1 isolate (ST124) belonged to a previously undesignated lineage (Table 2).

**Table 2:** Shared-type and lineage/sub-lineage distribution of 65 *M. tuberculosis* isolates from New Delhi, India.

Lineage/Sub-lineage	ST no.	No. of isolates	Total no. of isolates (%)
CAS1_Delhi	26	16	30 (46.2)
	25	5	
	141	4	
	381	2	
	1327	1	
	1343	1	
	1344	1	
EAI5	138	6	6 (9.2)
EAI6_BGD1	1409	4	4 (6.2)
EAI3_IND	11	4	4 (6.2)
CAS	357	1	4 (6.2)
	952	1	
	1264	1	
	1616	1	
T1	1877	2	4 (6.2)
	53	1	
	1233	1	
Beijing	1	3	3 (4.6)
CAS2	288	2	2 (3.1)
X1	1394	1	1 (1.5)
X2	137	1	1 (1.5)
Previously undescribed	Orphan	5	5 (7.7)
Previously undesignated	ST124	1	1 (1.5)

### Single Nucleotide Polymorphism Analysis

PCR and subsequent SNP analysis was performed on 65 isolates for the genes *katG* ^364^ and *gyrA* ^95^. Eight isolates (12.3%) had a point-mutation at position *katG* ^463^, whereas no mutations were observed for *gyrA* ^95^ and hence, the isolates were assigned to PGG2. Six of the 8 isolates with a mutation in *katG* ^463^ belonged to the T1 (n = 4), X1 (n = 1) and X2 (n = 1) sub-lineages, whereas 2 isolates belonged to previously undescribed lineages. All other isolates (87.7%) analyzed were assigned to the genogroup PGG1 (Figure 1, Table 3). SNP analysis was also performed for 4 genes know to be involved in resistance to the anti-TB drugs isoniazid [INH] (*katG*), rifampicin [RIF] (*rpoB*), ethambutol (*embB*) and pyrazinamide [PZA] (*pncA*). The point-mutations observed for all 4 genes are described in Table 3. Thirty-two isolates had a mutation in at least the *rpoB* gene of which 23 had combined *katG* and *rpoB* mutations (MDR-TB). Of the 23 MDR-TB isolates, 11 belonged to the CAS1_DELHI sub-lineage, 3 to the Beijing lineage, 3 to the EAI3_IND sub-lineage, 2 to the CAS lineage, 2 to the EAI5 sub-lineage, 1 to the CAS2 sub-lineage, and 1 to the X2 sub-lineage. Nine isolates had combined mutations in the *katG*, *rpoB* and *embB* genes. Three isolates had a mutation in the *katG* gene only, whereas 1 isolate had combined *katG* and *embB* mutations. Of the 10 isolates with a mutation in the *pncA* gene conferring resistance to PZA, 7 also had mutations in both *rpoB* and *katG*, 1 isolate had mutations in *rpoB* and *embB*, whereas the 2 remaining isolates with a *pncA* mutation were wild-type for the other 3 genes analysed. One isolate had a mutation in the *embB* gene only.

**Table 3:** Number of single nucleotide polymorphisms observed for the 6 regions of interest in 65 *M. tuberculosis* isolates from New Delhi, India.

Gene	Mutation	No. of isolates with mutation
*katG*	G->C pos 944, S315T	22
	G->A pos 944, S315N	3
	G->A pos 836, G279D	1
	G->A pos 946, R316Q	1
	A->C pos 970, T324P	1
*rpoB*	C->T pos 1350, S531L	12
	A->G pos 1335, H526D	4
	A->T pos 1305, D516V	4
	C->T pos 1334, H526D	3
	G->T pos 1304, D516V	3
	T->C pos 1356, L533P	2
	C->A pos 1334, H526N	1
	C->G pos 1334, H526D	1
	C->G pos 1350, S531L	1
	C->A pos 1273, F505L	1
	T->C pos 1290, L511P	1
	C->A pos 1295, Q513K	1
*embB*	A->G pos 917, M306I	6
	G->A pos 919, M306I	3
	A->C pos 917, M306L	2
	A->C pos 957, Y319S	1
	G->A pos 949, M316I	1
	G->C pos 919, M306I	1
*pncA*	C->T pos 195, S65S	35
	A->C pos 535, S178R	2
	A->C pos 35, D12A	1
	Del C pos 344, STOP116	1
	Del G pos 166, STOP116	1
	Ins G pos 234, STOP126	1
	Ins G pos 394, Frameshift	1
	T->C pos 104, L34P	1
	G->C pos 357, W119C	1
	G->C pos 481, A160P	1
*katG^463^*	CTG->CGG pos 463	8
*gyrA^95^*	No mutations observed	-

The most frequently observed mutation was a silent C->T mutation in codon 65 of the *pncA* gene. This mutation was observed in all but two isolates of the CAS lineage and in 1 orphan, whereas none of the other isolates had this mutation (Table 3, Figure 1). The most frequently observed mutation in *katG* was a G->C substitution at position 944, resulting in an S315T substitution in 22 isolates. Twelve isolates had a C->T mutation in position 1350 of *rpoB*, resulting in an S531L substitution, whereas 6 isolates had an A->G mutation at position 917 in *embB*, causing an M306I substitution (Table 3).

Mutations in the *rpoB* gene, the *katG* gene, and in both *rpoB* and *katG* genes were together more frequent in CAS1_DELHI isolates compared to non-CAS_DELHI isolates (OR: 3.1, CI95% [1.11, 8.70], P = 0.045). However, the increased frequency of drug-resistance observed for the CAS1_DELHI sub-lineage isolates was not linked the patients' history of previous anti-TB treatment (OR: 1.156, CI95% [0.40, 3.36], *P* = 0.79).

#### TbD1 analysis

All isolates were analyzed for the presence or absence of the TbD1 region by PCR. Seventeen isolates belonging to EAI5 (ST38, n = 6), EAI6_BGD1 (ST1409, n = 4), EAI3_IND (ST11, n = 4) and three orphans belonging to previously undescribed lineages had this region intact, whereas all other isolates lacked this region. All isolates with an intact TbD1 belonged to the PGG1 genogroup (Figure 1).

## Discussion

Genomic plasticity and phenotypic versatility of pathogenic bacteria are the two major forces that stratify potentially virulent and benign types [25]. Our understanding of mycobacterial evolution is partly based on this paradigm where pathogenic ‘specialist’ forms are thought to have evolved from their ‘generalist’ predecessors [26]. While globally, the core gene pools of such bacteria are highly clonal [21], there may be certain plastic regions that experience frequent genomic recasting [22]. Accurate analysis of the repertoire of such elements in a given population based on multiple genome profiling approaches could reveal significant aspects of ongoing evolution (and transmission) and the available magnitude of genetic diversity among the strains could thus be unraveled. This ultimately helps one understand transmission cum virulence potentials of strains and should ideally constitute an important agenda for epidemic control programs.

*M. tuberculosis* strains of the so called PGG1 [21] have been prevalent in India with CAS (TbD1^−^) and EAI (TbD1^+^) being the major genogroups. While EAI strains have been the focus of attention for some time owing to their presumed ‘low virulent’ characteristics [27], [28] and their links to ancestral African predecessors [21], [29], the origins and routes of the spread of the CAS strains are not clear. Although these lineages are prevalent in countries of Central- and West-Asian regions it is not evident why they are specifically predominant in the Delhi region. Despite the characteristic deletion of the TbD1 region, CAS strains have never been linked to any institutionalized outbreaks as seen for example in the case of the TbD1^−^ Beijing-lineage isolates [3], [30]. The spread of the CAS strains through the western corridors and subsequent adaptation to the North Indian populations is an imminent possibility. However, more in-depth analyses involving multiple genetic loci are essential in order to delineate the origin and spread route of the CAS strains. Our study was partly undertaken with this objective.

The genotypic analyses employed in this study showed that the majority of isolates (73.8%) belonged to the TbD1^−^ PGG1 genogroup, whereas only 26.2% belonged to lineages with an intact TbD1 region. The relatively low number of TbD1^+^ isolates observed in this study is in line with previous studies from other parts of North India, which show a predominance of TbD1^−^ strains in the North, whereas Central- and South India are dominated by TbD1^+^ strains [2], [7], [14], [18], [31]. Spoligotyping results from this study confirmed previous studies [5], [18], [32] which show that the CAS1_DELHI sub-lineage dominates in the Delhi region and that ST26 is one of the most frequently observed genotypes in this area.

Strains of the ST1 genotype (Beijing lineage) are reportedly the most prevalent genotype world-wide [16]. In contrast to other urban areas in South-East Asia [33], for this study, only 4.6% (n = 3) of the isolates belonged to the ST1 genotype compared to 24.6% (n = 16) of ST26 genotype. The low frequency of Beijing isolates in this region is in concordance with other studies from Karachi, Pakistan [34] and Delhi, India [7]. Like isolates of the hypervirulent and MDR-associated ST1 genotype, the ST26 isolates belong to the TbD1^−^ PGG1 genogroup. Isolates of the Beijing lineage have been shown to be less prevalent in North India compared to the central and southern parts [18]. All ST1 isolates and 31.3% of ST26 isolates in this study had mutations in at least the *rpoB* and *katG* genes. Unlike the higher frequency of MDR reported for isolates belonging to the ST1 genotype, no significant increase in the frequency of combined mutations in the *rpoB* and *katG* genes in the ST26 genotype could be established. Furthermore, in agreement with previously published results [4], [34], isolates belonging to the CAS1 sub-lineages did not show an increased rate of MDR. However, our study showed that mutations conferring resistance to both RIF and INH, key first-line anti-TB drugs, were more frequent in CAS1_DELHI isolates compared to non-CAS_DELHI isolates (OR: 3.1, P = 0.045). Mutations (deletions and insertions) in the *rpoB* and *katG* genes are considered to be synonymous with resistance to RIF [35] and INH [36], respectively. The estimated frequency of spontaneous mutations conferring resistance to INH are 3.5×10^−6^ and 3.1×10^−8^ for RIF [37]. The risk of a double spontaneous mutation is very low, estimated to occur at a frequency of 9×10^−14^ [38]. For this study, mutations in the *rpoB* gene were observed in 34 out of 65 (52.3%) of the isolates, of which 79.4% (n = 27) of the *M. tuberculosis* isolates belonged to TbD1− genotypes. Sixty percent of the isolates with a mutation in the *rpoB* gene belonged to the CAS1_DELHI sub-lineage. The MIRU-VNTR results clearly show that although similar (26 of 36 CAS isolates had >90% similarity in their MIRU-VNTR types), the isolates were not clones from a recent outbreak. There was no apparent association between patients having received previous treatment for TB and mutations conferring drug-resistance to RIF and INH for CAS1_DELHI sub-lineages, suggesting that there may be a higher frequency of circulating drug-resistant CAS1_DELHI isolates in New Delhi.

Other genotypic studies from India [7], [18] are based solely on spoligotypes. In our study, high resolution genotyping, based on the multiple markers employed, was able to provide clues with regard to the evolutionary history of the CAS sub-lineages. The secondary sequence typing approach (*gyrA*/*katG* and *pncA* genes) revealed a CAS-lineage specific silent C->T mutation in codon 65 of the *pncA* gene. This mutation was present in all observed ST's within the CAS lineage, except for 2 out of 16 ST26 (CAS1_DELHI) isolates, suggesting that the mutating lineages are descendents of the ancestral CAS1_DELHI type. Previous studies [39]–[41] show that there does not seem to be any association between this mutation and resistance to PZA. In order to determine if this mutation is generalizable to CAS isolates from other geographical locations, isolates from Myanmar (n = 15) and South Africa (n = 4) were also analyzed. All isolates from South Africa and Myanmar, except two isolates from Myanmar belonging to the ST26 and ST1378 genotypes, had the C->T mutation in codon 65 of the *pncA* gene. This mutation could potentially be utilized to develop a rapid CAS specific PCR-based genotyping assay, which could be useful in phylogeographical studies and in epidemiological investigations of TB outbreaks.

In addition to the silent mutation in codon 65 of the *pncA* gene, 9 other point-mutations in the *pncA* gene were observed (Figure 1 and Table 3). The *pncA* gene codes for the enzyme pyrazinamidase, which converts the pro-drug PZA, to its active form. PZA is one of the cornerstone drugs retained in the treatment of MDR-TB. Previous studies [42]–[44] have shown that mutations in the structural gene or promoter region of *pncA*, leading to an amino acid substitution, confer resistance to PZA in 72–97% of the cases. We have recently shown that a high proportion of South African MDR *M. tuberculosis* isolates are resistant to PZA [42]. In this study, we show that 30.4% of the MDR isolates were also resistant to PZA. Further studies with systematic testing for PZA are required to assess if these findings are representative for MDR isolates from other states in India.

Careful monitoring of circulating strains is required to detect new and emerging genotypes. Isolates belonging to the CAS1_DELHI sub-lineage should be fully characterized with regards to their level of virulence, transmissibility and mutability to determine if they share certain characteristics with the hypervirulent and highly transmissible Beijing lineage. The association between the CAS1_DELHI isolates and drug-resistance observed for this study may be attributed to a low sample size and the fact that the isolates were collected from a single hospital in an urban setting. Thus larger studies, including isolates from more remote areas, are required in order to fully delineate the association of the CAS1_DELHI isolates with drug-resistance. Additionally, screening for the *pncA* S65S mutation in isolates from other shared-types of the CAS-lineage may aid in the reconstruction of the phylogenetic relationships of genotypes within this lineage.

## References

[1] WHO (2008). WHO Report 2008..

[2] Gutierrez MC, Ahmed N, Willery E, Narayanan S, Hasnain SE (2006). Predominance of ancestral lineages of *Mycobacterium tuberculosis* in India.. Emerg Infect Dis.

[3] Almeida D, Rodrigues C, Ashavaid TF, Lalvani A, Udwadia ZF (2005). High incidence of the Beijing genotype among multidrug-resistant isolates of *Mycobacterium tuberculosis* in a tertiary care center in Mumbai, India.. Clin Infect Dis.

[4] Hasan Z, Tanveer M, Kanji A, Hasan Q, Ghebremichael S (2006). Spoligotyping of *Mycobacterium tuberculosis* isolates from Pakistan reveals predominance of Central Asian Strain 1 and Beijing isolates.. J Clin Microbiol.

[5] Kulkarni S, Sola C, Filliol I, Rastogi N, Kadival G (2005). Spoligotyping of *Mycobacterium tuberculosis* isolates from patients with pulmonary tuberculosis in Mumbai, India.. Res Microbiol.

[6] Siddiqi N, Shamim M, Amin A, Chauhan DS, Das R (2001). Typing of drug resistant isolates of *Mycobacterium tuberculosis* from India using the IS*6110* element reveals substantive polymorphism.. Infect Genet Evol.

[7] Singh UB, Suresh N, Bhanu NV, Arora J, Pant H (2004). Predominant tuberculosis spoligotypes, Delhi, India.. Emerg Infect Dis.

[8] Oelemann MC, Diel R, Vatin V, Haas W, Rusch-Gerdes S (2007). Assessment of an optimized mycobacterial interspersed repetitive- unit-variable-number tandem-repeat typing system combined with spoligotyping for population-based molecular epidemiology studies of tuberculosis.. J Clin Microbiol.

[9] van Embden JD, Cave MD, Crawford JT, Dale JW, Eisenach KD (1993). Strain identification of *Mycobacterium tuberculosis* by DNA fingerprinting: recommendations for a standardized methodology.. J Clin Microbiol.

[10] Kremer K, van Soolingen D, Frothingham R, Haas WH, Hermans PW (1999). Comparison of methods based on different molecular epidemiological markers for typing of *Mycobacterium tuberculosis* complex strains: interlaboratory study of discriminatory power and reproducibility.. J Clin Microbiol.

[11] van Soolingen D, de Haas PE, Hermans PW, Groenen PM, van Embden JD (1993). Comparison of various repetitive DNA elements as genetic markers for strain differentiation and epidemiology of *Mycobacterium tuberculosis*.. J Clin Microbiol.

[12] Braden CR, Crawford JT, Schable BA (2002). Quality assessment of *Mycobacterium tuberculosis* genotyping in a large laboratory network.. Emerg Infect Dis.

[13] Hanekom M, van der Spuy GD, Gey van Pittius NC, McEvoy CR, Hoek KG (2008). Discordance between mycobacterial interspersed repetitive-unit-variable-number tandem-repeat typing and IS*6110* restriction fragment length polymorphism genotyping for analysis of *Mycobacterium tuberculosis* Beijing strains in a setting of high incidence of tuberculosis.. J Clin Microbiol.

[14] Narayanan S, Gagneux S, Hari L, Tsolaki AG, Rajasekhar S (2008). Genomic interrogation of ancestral *Mycobacterium tuberculosis* from south India.. Infect Genet Evol.

[15] Sharma P, Chauhan DS, Upadhyay P, Faujdar J, Lavania M (2008). Molecular typing of *Mycobacterium tuberculosis* isolates from a rural area of Kanpur by spoligotyping and mycobacterial interspersed repetitive units (MIRUs) typing.. Infect Genet Evol.

[16] Brudey K, Driscoll JR, Rigouts L, Prodinger WM, Gori A (2006). *Mycobacterium tuberculosis* complex genetic diversity: mining the fourth international spoligotyping database (SpolDB4) for classification, population genetics and epidemiology.. BMC Microbiol.

[17] Gagneux S, Small PM (2007). Global phylogeography of *Mycobacterium tuberculosis* and implications for tuberculosis product development.. Lancet Infect Dis.

[18] Singh UB, Arora J, Suresh N, Pant H, Rana T (2007). Genetic biodiversity of *Mycobacterium tuberculosis* isolates from patients with pulmonary tuberculosis in India.. Infect Genet Evol.

[19] Dale JW, Brittain D, Cataldi AA, Cousins D, Crawford JT (2001). Spacer oligonucleotide typing of bacteria of the *Mycobacterium tuberculosis* complex: recommendations for standardised nomenclature.. Int J Tuberc Lung Dis.

[20] Supply P, Allix C, Lesjean S, Cardoso-Oelemann M, Rusch-Gerdes S (2006). Proposal for standardization of optimized mycobacterial interspersed repetitive unit-variable-number tandem repeat typing of *Mycobacterium tuberculosis*.. J Clin Microbiol.

[21] Sreevatsan S, Pan X, Stockbauer KE, Connell ND, Kreiswirth BN (1997). Restricted structural gene polymorphism in the *Mycobacterium tuberculosis* complex indicates evolutionarily recent global dissemination.. Proc Natl Acad Sci U S A.

[22] Brosch R, Gordon SV, Marmiesse M, Brodin P, Buchrieser C (2002). A new evolutionary scenario for the *Mycobacterium tuberculosis* complex.. Proc Natl Acad Sci U S A.

[23] Hunter PR, Gaston MA (1988). Numerical index of the discriminatory ability of typing systems: an application of Simpson's index of diversity.. J Clin Microbiol.

[24] Sola C, Filliol I, Legrand E, Lesjean S, Locht C (2003). Genotyping of the *Mycobacterium tuberculosis* complex using MIRUs: association with VNTR and spoligotyping for molecular epidemiology and evolutionary genetics.. Infect Genet Evol.

[25] Ahmed N, Dobrindt U, Hacker J, Hasnain SE (2008). Genomic fluidity and pathogenic bacteria: applications in diagnostics, epidemiology and intervention.. Nat Rev Microbiol.

[26] Ahmed N, Saini V, Raghuvanshi S, Khurana JP, Tyagi AK (2007). Molecular analysis of a leprosy immunotherapeutic bacillus provides insights into Mycobacterium evolution.. PLoS ONE.

[27] Ahmed N, Ehtesham NZ, Hasnain SE (2008). Ancestral *Mycobacterium tuberculosis* genotypes in India: implications for TB control programmes.. Infect Genet Evol.

[28] Ahmed N, Leblebicioglu H (2006). India's ‘gold mine’ of ancestral bacilli and the looming TB-HIV pandemic.. Ann Clin Microbiol Antimicrob.

[29] Gutierrez MC, Brisse S, Brosch R, Fabre M, Omais B (2005). Ancient origin and gene mosaicism of the progenitor of *Mycobacterium tuberculosis*.. PLoS Pathog.

[30] Agerton T, Valway S, Gore B, Pozsik C, Plikaytis B (1997). Transmission of a highly drug-resistant strain (strain W1) of *Mycobacterium tuberculosis*. Community outbreak and nosocomial transmission via a contaminated bronchoscope.. Jama.

[31] Bhanu NV, van Soolingen D, van Embden JD, Dar L, Pandey RM (2002). Predominace of a novel *Mycobacterium tuberculosis* genotype in the Delhi region of India.. Tuberculosis (Edinb).

[32] Suresh N, Singh UB, Arora J, Pant H, Seth P (2006). rpoB gene sequencing and spoligotyping of multidrug-resistant *Mycobacterium tuberculosis* isolates from India.. Infect Genet Evol.

[33] Rahim Z, Zaman K, van der Zanden AG, Mollers MJ, van Soolingen D (2007). Assessment of population structure and major circulating phylogeographical clades of *Mycobacterium tuberculosis* complex in Bangladesh suggests a high prevalence of a specific subclade of ancient M. tuberculosis genotypes.. J Clin Microbiol.

[34] Tanveer M, Hasan Z, Siddiqui AR, Ali A, Kanji A (2008). Genotyping and drug resistance patterns of M. tuberculosis strains in Pakistan.. BMC Infect Dis.

[35] Telenti A, Imboden P, Marchesi F, Lowrie D, Cole S (1993). Detection of rifampicin-resistance mutations in *Mycobacterium tuberculosis*.. Lancet.

[36] Zhang Y, Heym B, Allen B, Young D, Cole S (1992). The catalase-peroxidase gene and isoniazid resistance of *Mycobacterium tuberculosis*.. Nature.

[37] Tsukamura M (1972). The pattern of resistance development to rifampicin in *Mycobacterium tuberculosis*.. Tubercle.

[38] Dooley S, Simone M, Davis PDO (1994). The extent and management of drug-resistant tuberculosis: the American experience. In: Clinical Tuberculosis..

[39] Jureen P, Werngren J, Toro JC, Hoffner S (2008). Pyrazinamide resistance and *pncA* gene mutations in *Mycobacterium tuberculosis*.. Antimicrob Agents Chemother.

[40] Sekiguchi J, Nakamura T, Miyoshi-Akiyama T, Kirikae F, Kobayashi I (2007). Development and evaluation of a line probe assay for rapid identification of *pncA* mutations in pyrazinamide-resistant *Mycobacterium tuberculosis* strains.. J Clin Microbiol.

[41] Somoskovi A, Dormandy J, Parsons LM, Kaswa M, Goh KS (2007). Sequencing of the pncA gene in members of the *Mycobacterium tuberculosis* complex has important diagnostic applications: Identification of a species-specific *pncA* mutation in “Mycobacterium canettii” and the reliable and rapid predictor of pyrazinamide resistance.. J Clin Microbiol.

[42] Mphahlele M, Syre H, Valvatne H, Stavrum R, Mannsaker T (2008). Pyrazinamide resistance among South African multidrug-resistant *Mycobacterium tuberculosis* isolates.. J Clin Microbiol.

[43] Scorpio A, Lindholm-Levy P, Heifets L, Gilman R, Siddiqi S (1997). Characterization of *pncA* mutations in pyrazinamide-resistant *Mycobacterium tuberculosis*.. Antimicrob Agents Chemother.

[44] Sreevatsan S, Pan X, Zhang Y, Kreiswirth BN, Musser JM (1997). Mutations associated with pyrazinamide resistance in *pncA* of *Mycobacterium tuberculosis* complex organisms.. Antimicrob Agents Chemother.

[45] Silva MS, Senna SG, Ribeiro MO, Valim AR, Telles MA (2003). Mutations in *katG*, *inhA*, and *ahpC* genes of Brazilian isoniazid-resistant isolates of *Mycobacterium tuberculosis*.. J Clin Microbiol.

[46] De Beenhouwer H, Lhiang Z, Jannes G, Mijs W, Machtelinckx L (1995). Rapid detection of rifampicin resistance in sputum and biopsy specimens from tuberculosis patients by PCR and line probe assay.. Tuber Lung Dis.

[47] Victor TC, Jordaan AM, van Rie A, van der Spuy GD, Richardson M (1999). Detection of mutations in drug resistance genes of *Mycobacterium tuberculosis* by a dot-blot hybridization strategy.. Tuber Lung Dis.

